# Twinned growth behaviour of two-dimensional materials

**DOI:** 10.1038/ncomms13911

**Published:** 2016-12-20

**Authors:** Tao Zhang, Bei Jiang, Zhen Xu, Rafael G. Mendes, Yao Xiao, Linfeng Chen, Liwen Fang, Thomas Gemming, Shengli Chen, Mark H. Rümmeli, Lei Fu

**Affiliations:** 1 College of Chemistry and Molecular Sciences, Wuhan University, Wuhan 430072, China; 2 Leibniz Institute for Solid State and Materials Research Dresden, P.O. Box 270116, Dresden D-01171, Germany; 3 College of Physics, Optoelectronics and Energy & Collaborative Innovation Center of Suzhou Nano Science and Technology, Soochow University, Suzhou 215006, China; 4 Centre of Polymer and Carbon Materials, Polish Academy of Sciences, M. Curie-Sklodowskiej 34, Zabrze 41-819, Poland

## Abstract

Twinned growth behaviour in the rapidly emerging area of two-dimensional nanomaterials still remains unexplored although it could be exploited to fabricate heterostructure and superlattice materials. Here we demonstrate how one can utilize the twinned growth relationship between two two-dimensional materials to construct vertically stacked heterostructures. As a demonstration, we achieve 100% overlap of the two transition metal dichalcogenide layers constituting a ReS_2_/WS_2_ vertical heterostructure. Moreover, the crystal size of the stacked structure is an order of magnitude larger than previous reports. Such twinned transition metal dichalcogenides vertical heterostructures exhibit great potential for use in optical, electronic and catalytic applications. The simplicity of the twinned growth can be utilized to expand the fabrication of other heterostructures or two-dimensional material superlattice and this strategy can be considered as an enabling technology for research in the emerging field of two-dimensional van der Waals heterostructures.

## Abstract (web summary)

Precise control of the growth of transition metal dichalcogenides layers in atomically thin van der Waals heterostructures has gained significant attention. Here, the authors report 100% overlapped vertical ReS_2_/WS_2_ heterostructures by utilizing the twinned growth behaviour of two-dimensional materials.

## Graphical abstract

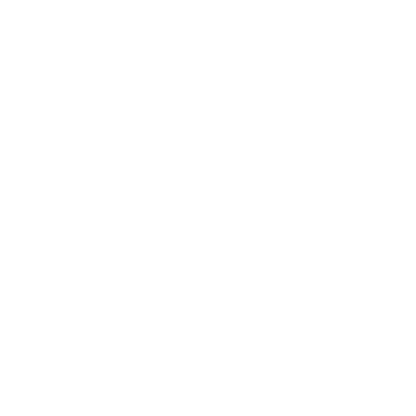

The rational stacking of two different transition metal dichalcogenides (TMDCs, two-dimensional atomic crystalline materials with tunable electronic structure^1^,^2^,^3^,^4^,^5^ and great potential in optoelectronic devices^6^,^7^,^8^,^9^,^10^), has drawn significant attention as it endows TMDCs with great opportunities to expand their pristine properties^11^,^12^,^13^,^14^,^15^ and broaden their applications^16^,^17^,^18^,^19^,^20^. Initially, vertically stacked heterostructures comprising multi-layered TMDCs were obtained by sequential mechanical exfoliation. These studies triggered subsequent studies focused on the top-down creation of TMDCs heterostructures with varied chemical composition, interlayer spacing, and angular alignment^11^. However, due to the limited size and randomly generated locations of the exfoliated TMDCs flakes, this stacking procedure is neither controllable nor scalable. Later, Ajayan *et al*. presented an approach for the chemical vapour deposition (CVD) synthesis of stacked WS_2_/MoS_2_ heterostructures, which showed the potential for larger scale production^21^. However, due to the random nucleation process and poor control over the growth rate, it is extremely difficult to significantly increase the stacking area or to precisely control the growth behaviour of vertically stacked TMDCs heterostructures. Thus far, the stacking of two TMDCs constituents in a strictly controlled manner (especially in terms of control over the overlap percentage) has yet to be achieved, even though this is highly desirable.

Herein, we demonstrate the twinned growth of two 2D (two-dimensional) nanomaterials, namely ReS_2_ and WS_2_. We achieve 100% overlap for each of the stacked TMDCs structures, with crystal sizes of the heterostructures one order of magnitude larger than previous reports^12^,^13^,^14^,^16^,^18^,^20^,^21^,^22^,^23^,^24^,^25^,^26^. For the twinned growth of ReS_2_/WS_2_ vertical heterostructures, Au is chosen as the substrate and W–Re alloy foil is used as the Re and W sources, which lower the barrier energies for this special twinned growth process. We believe that the developed approach will promote and accelerate ongoing research efforts of 2D crystalline van der Waals heterostructures.

## Results

### The morphology and spectral characterization of ReS_2_/WS_2_

**Figure 1:**
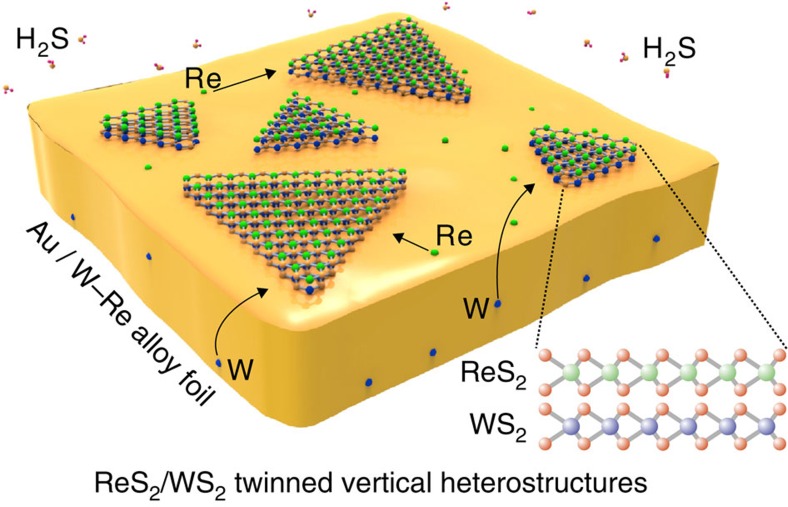
Schematic for the twinned growth of ReS_2_/WS_2_. For the growth of ReS_2_/WS_2_ twinned vertical heterostructures, Au is chosen as the growth substrate and W–Re alloy foil serves as the support substrate. This twinned growth process starts upon the introduction of H_2_S.

**Figure 2:**
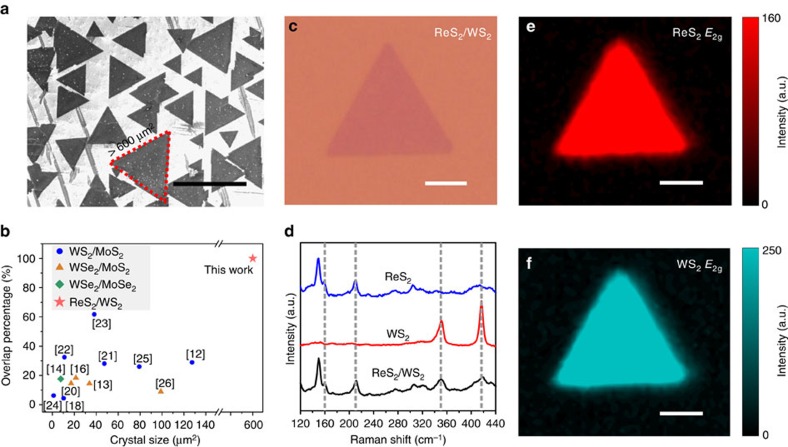
Characterizations of ReS_2_/WS_2_ twinned vertical heterostructures. (**a**) SEM image of the triangular ReS_2_/WS_2_ vertical crystalline heterostructures over Au. A ReS_2_/WS_2_ crystal with grain size up to 600 μm^2^ is indicated. (**b**) Crystal size plotted as a function of overlap percentage of TMDCs heterostacks obtained in this work and those reported in other literature. (**c**) Optical image of a ReS_2_/WS_2_ twinned vertical heterostructure crystal after transfer onto a Si/SiO_2_ substrate. (**d**) Comparison of the Raman spectra of ReS_2_, WS_2_, and ReS_2_/WS_2_, in which four distinct peaks are observed from the twinned ReS_2_/WS_2_ crystal (**e**,**f**) Raman mappings of peak intensity at 160.5 and 351.3 cm^−1^ respectively, corresponding to the *E*_2g_ mode of ReS_2_ and WS_2_ respectively. Scale bar, 40 μm (**a**); 5 μm (**c**,**e**,**f**).

Figure 1 schematically shows the strategy for the twinned growth of ReS_2_ and WS_2_ towards vertically stacked heterostructures. From scanning electron microscopy (SEM) images (for example, Fig. 2a) taken from the Au surface after the CVD reaction, one can observe large-area uniformity and a high yield of triangular vertically stacked ReS_2_/WS_2_ crystals. One can also see that the domain size of the smaller stacked ReS_2_/WS_2_ structures is typically >10 μm, while the bigger crystal reaches a large size of 600 μm^2^ (Fig. 2a). This is about 1 order of magnitude larger than other reports of vertically stacked TMDCs heterostructures synthesized by traditional CVD technique or by a sequential exfoliation process (Fig. 2b). Moreover, from Fig. 2b, we can also conclude that a 100% overlap of two TMDCs is obtained, and this is also much higher than previously reported findings. To further characterize the structure of the twinned ReS_2_/WS_2_ crystals, the samples were transferred onto 300 nm Si/SiO_2_ substrates. After transfer, typical optical image of the triangular crystal is shown as Fig. 2c, which reveals that the stacked ReS_2_/WS_2_ structure exhibits a uniform light-purple triangle consistent with a high uniformity in agreement with the SEM data (representative image can be seen in Fig. 2a). In addition, atomic force microscope images of the transferred crystals show a uniform height of ∼1.6 nm over the entire structure (Supplementary Fig. 1), which confirms the bilayered, 100% overlapped structure and twinned growth nature of the ReS_2_/WS_2_ heterostructures.

Figure 2d shows the comparison between the Raman spectra of pure ReS_2_, pure WS_2_ and the twinned ReS_2_/WS_2_ heterostructure. Within the Raman spectrum of heterostructure, one can observe peaks from both ReS_2_ (*E*_2g_≈160.5 cm^−1^ and *A*_1g_≈211.3 cm^−1^)^27^ and WS_2_ (*E*_2g_≈351.3 cm^−1^ and *A*_1g_≈417.8 cm^−1^)^28^. This shows that the structures are ReS_2_/WS_2_ vertically stacked heterostructures and not an alloyed Re_*x*_W_1*−x*_S_2_ crystal, since the Raman spectrum of a Re_*x*_W_1*−x*_S_2_ alloy would show two main peaks with their positions between the *E*_2g_ and *A*_1g_ peaks of pure ReS_2_ or WS_2_. To further confirm the 100% overlapped structure of the vertical heterostructures, Raman mapping was used. Typical examples are provided in Fig. 2e,f, in which Raman intensity maps using the ReS_2_ *E*_2g_ mode at 160.5 cm^−1^ and the WS_2_ *E*_2g_ mode at 351.3 cm^−1^ demonstrate a uniform response for both ReS_2_ and WS_2_ over the entire crystal. This rules out the possibility of alloying. In addition, Raman spectra were collected at fixed spatial positions at the center and corners of 11 randomly selected triangle ReS_2_/WS_2_ crystals. Each verified the distinct signatures corresponding to ReS_2_ and WS_2_ (Supplementary Fig. 2), so that the 100% overlapped structure and the large-scale twinned growth of the ReS_2_/WS_2_ vertical heterostructures were further confirmed.

### Optoelectronic characterization of ReS_2_/WS_2_

**Figure 3:**
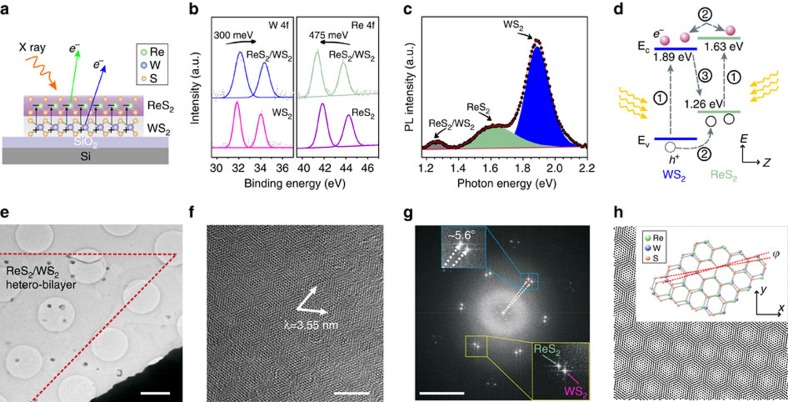
Structure characterization of ReS_2_/WS_2_ twinned vertical heterostructures. (**a**) Sketch of the X-ray photoelectron analyses of ReS_2_/WS_2_ heterostack. (**b**) XPS core level shift analyses of ReS_2_/WS_2_ heterostructures. (**c**) Photoluminescence spectrum of the ReS_2_/WS_2_ heterostack, in which the three peaks are attributed to the exciton relaxations within ReS_2_ (green), WS_2_ (blue) and between ReS_2_/WS_2_ layers (grey). (**d**) Band diagram of ReS_2_/WS_2_ heterostructures under photo excitation, depicting (1) absorption and exciton generation in WS_2_ and ReS_2_, (2) relaxation of excitons at the ReS_2_/WS_2_ interface driven by the band offset, and (3) radiative recombination of spatially indirect excitons. (**e**) Low-magnification TEM image of the ReS_2_/WS_2_ twinned vertical heterostructures, where a triangle crystal of ReS_2_/WS_2_ hetero-bilayer is marked. (**f**) High-resolution TEM image of ReS_2_/WS_2_ vertical heterostructures showing the resulting Moiré pattern. (**g**) Fast Fourier transform (FFT) of the heterostructures in **f**. The inset shows the two patterns of ReS_2_ (green) and WS_2_ (purple) with a rotation angle to be about 5.6°. (**h**) Tentative orientation model of rotating the upper ReS_2_ by an angle of 5.6° with respect to the ground WS_2_. Insert is the atomistic illustration of the heterostructure of ReS_2_/WS_2_ with their respective lattice constants and a misalignment angle *ϕ*. Scale bar, 1 μm (**e**); 4 nm (**f**); 4 nm^−1^ (**g**).

To explore the electronic structure of our ReS_2_/WS_2_ twinned heterostructures, X-ray photoelectron spectroscopy (XPS) was implemented (see Fig. 3a and Supplementary Fig. 3). From the XPS data, the (Re and W):S atomic ratio is 1:2.0, indicating that the twinned ReS_2_/WS_2_ is stoichiometric. As shown in Fig. 3b, comparison of the W 4f core level doublet from WS_2_ and ReS_2_/WS_2_ shows an up-shift of 300 meV, corresponding to a positive net charge on the WS_2_ bottom layer^29^. Similarly, comparison of the Re 4f core level doublet from ReS_2_ and ReS_2_/WS_2_ shows a down-shift of 475 meV, corresponding to a negative net charge on the ReS_2_ top layer^30^. Our results therefore indicate that the ReS_2_ layer has a negative net charge, while the WS_2_ layer has a positive net charge as a result of a contact potential. Hence the ReS_2_/WS_2_ heterostructures serve as an atomically thin capacitor with a potential up to 775 meV, which originates from the work function difference induced charge transfer between the constituent ReS_2_ and WS_2_ layers and is twice that of previously reported TMDCs vertical heterostructures^20^.

Photoluminescence (PL) spectrometry was used to investigate the interaction between ReS_2_/WS_2_ heterostack layers. As shown in Fig. 3c, three prominent peaks are observed at exciton transition energies of 1.89, 1.63 and 1.26 eV, corresponding to wavelengths of 657, 759 and 987 nm, respectively. The two strong exciton peaks at 1.63 eV (759 nm) and 1.89 eV (657 nm) are consistent with the PL intensities for ReS_2_ and WS_2_, and are in good agreement with previous works^29^,^30^. The weak peak at 1.26 eV (987 nm) can be attributed to an indirect exciton transition between the ReS_2_ and WS_2_ layers. This interlayer PL exciton transition is a type II band alignment, which highlights the twinned growth nature of the ReS_2_/WS_2_ layers forming the heterostructure. Figure 3d shows the band diagram for ReS_2_/WS_2_ heterostructures under photo excitation. Owing to energy lost to the band offset, the PL exciton peak energy (1.26 eV) is lower than the excitonic band gaps for either of the TMDCs constituents (1.63 eV for ReS_2_ or 1.89 eV for WS_2_). Moreover, the strong luminescence signal at energies corresponding to the excitonic band gaps of ReS_2_ (1.63 eV) and WS_2_ (1.89 eV) suggests that a minority of the photoexcited carriers are relaxed at the interface leading to a low luminescence signal from the spatially indirect recombination process. Although the intensity of the indirect excitonic peak is weak, it is still an indication that the interface of the twinned ReS_2_/WS_2_ is clean and contamination-free, which confirms the advantages of our strategy for the fabrication of high-quality TMDCs heterostructures.

### High-resolution characterization of ReS_2_/WS_2_

To further reveal the crystalline structure and the stack orientation of the twinned ReS_2_/WS_2_ heterostructures, high-resolution transmission electron microscopy (HRTEM) was utilized to characterize the samples after transferring them to a TEM grid or onto a Si/SiO_2_ substrate. Low-magnification TEM image of a ReS_2_/WS_2_ twinned vertical heterostructure is presented in Fig. 3e, in which the crystal edge is marked. The structural continuity within the ReS_2_/WS_2_ domain suggests that our twinned vertical heterostructure is of high quality. Clear Moiré pattern, with periodicity measured to be about 3.55 nm, is observed in the high-resolution TEM image of the stacked structures as shown in Fig. 3f and the corresponding result of Fast Fourier transform is exhibited in Fig. 3g. Inspection of the Fast Fourier transform pattern reveals that in this particular sample the two hexagonal reciprocal lattices are rotated by *ϕ*=5.6° with respect to each other and there is negligible strain in the two constituent layers. Further simulation of the atomic structure by rotating the upper ReS_2_ by the angle of *ϕ* with respect to the ground WS_2_ layer (Fig. 3h) has shown similar Moiré pattern to that observed in Fig. 3f. To further confirm the stacked nature of the heterostructure, cross-section TEM samples were prepared by focused ion beam (FIB). The cross-sectional TEM images of ReS_2_/WS_2_ heterostructure demonstrate the clean interface as well as the bilayer stacked structure of our twinned ReS_2_/WS_2_ heterostacks (Supplementary Fig. 4).

## Discussion

**Figure 4:**
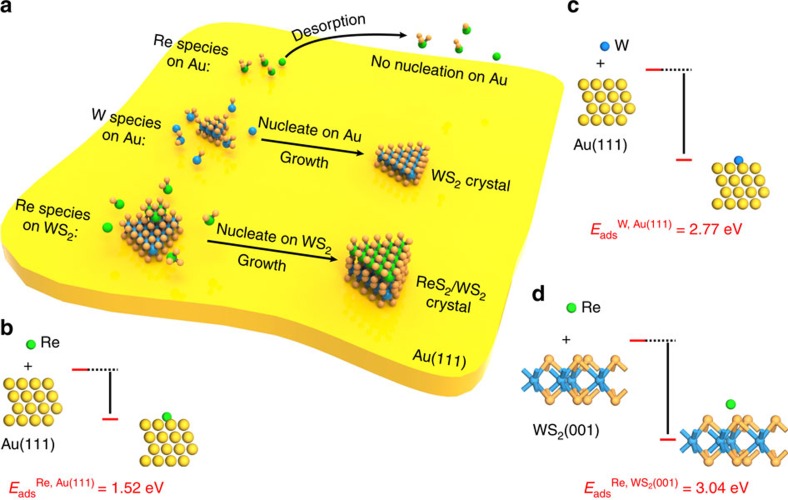
Theoretical simulations confirming the twinned growth nature between ReS_2_ and WS_2_. (**a**) Schematic illustrating the twinned growth of the ReS_2_/WS_2_ heterostructures over Au(111). (**b**–**d**) Side views of the simulated surface adsorption of Re atoms (**b**) and W atoms (**c**) over Au(111) and Re atoms over WS_2_(001) facet (**d**).

To have a better understanding of the twinned growth behaviour between the ReS_2_ and WS_2_ layers on Au, we simulated the growth process of ReS_2_ and WS_2_ based on the density functional theory (DFT) calculations (as shown in Fig. 4). Before these first-principles calculations, however, X-ray diffraction investigations were conducted. The data confirmed that the Au substrate is crystalline (Supplementary Fig. 5). When referenced with JCPDS data (JCPDS 04–0784), a clear match for Au (111) is obtained. Moreover, this was found for the entire surface, which confirms the single crystalline nature of our Au substrates.

Although the growth process of ReS_2_ and WS_2_ is complex, it can be simplified as follows^31^. The Re and W precursors are partially reduced by H_2_S gas to form a subsulfide species of ReS_*x*_ and WS_*x*_ (*x*=1∼3), which are then further sulfurized into ReS_2_ or WS_2_ over the substrate (as shown in Fig. 4a). To confirm the proposed mechanism, the adsorption energies for Re, W atoms over Au (111) and for Re atoms over WS_2_ (001) were calculated using first-principles calculations within DFT. The adsorption results for these atoms on the different substrates are provided in Fig. 4b–d. From the data it is clear that the adsorption energies for W atoms over Au(111) and Re atoms on WS_2_(001) are very close to each other (*E*_ads_^W, Au(111)^=2.77 eV, 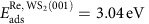). However, the adsorption energy for Re atoms over Au(111) (*E*_ads_^Re, Au(111)^=1.52 eV) is far weaker. At the implemented high growth temperature of 900 °C this weak energy would easily be overcome, resulting in the facile desorption of Re species making the nucleation of ReS_2_ highly unlikely. However, the strong adsorption energy (2.77 eV) is sufficiently accessible for WS_2_ to nucleate and grow over Au (111). Once WS_2_ forms on the substrate, the high adsorption energy of Re atom on WS_2_(001) would induce the subsequent adsorption, nucleation and growth of ReS_2_ on the newly formed WS_2_ surface. This beautifully highlights the twinned growth of ReS_2_/WS_2_ crystals.

**Figure 5:**
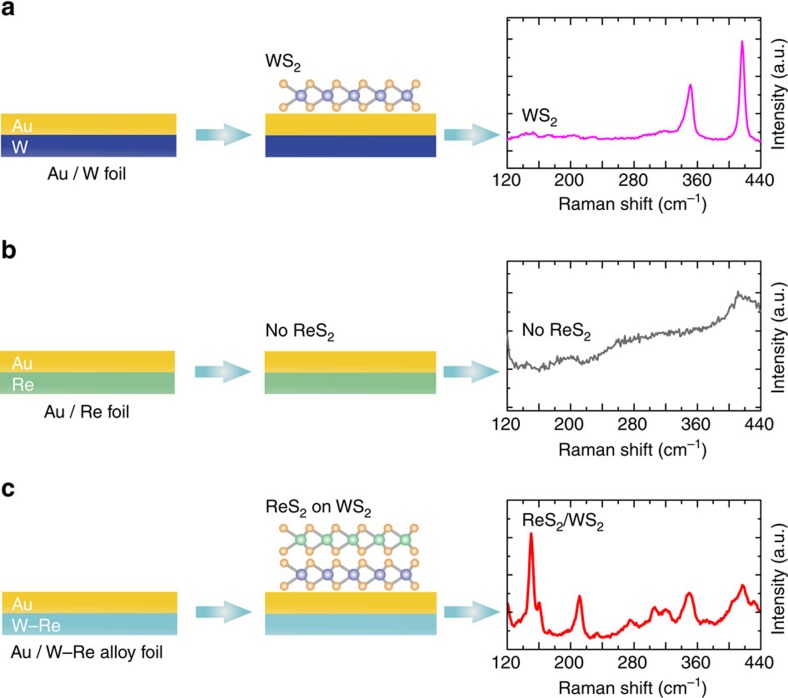
Scheme and Raman data illustrating the twinned growth nature between ReS_2_ and WS_2_. (**a**–**c**) Growth schematics and Raman spectra collected on Au while using W foil (**a**), Re foil (**b**) and W–Re alloy foil (**c**) as the support substrate.

To further confirm the twinned growth behaviour, different support substrates, namely, W foil, Re foil and W–Re alloy foil were investigated at a growth temperature of 900 °C. Figure 5 shows the scheme for the different support substrates and corresponding growth results. When W foil is used, WS_2_ can easily form on the surface of Au(111) (see Fig. 5a and Supplementary Fig. 6), confirming the high adsorption energy of W atoms on Au(111). This allows the nucleation and growth of WS_2_ on Au(111) at 900 °C. However, when Re foil is used as the support substrate, no ReS_2_ Raman response is found on the Au(111) surface (as shown in Fig. 5b). This indicates that ReS_2_ is not obtained at 900 °C due to the facile desorption of Re atoms from Au(111) because of their low adsorption energy. However, when the W–Re alloy was used as the support substrate, four distinct peaks arising from the *E*_2g_, *A*_1g_ modes of both ReS_2_ and WS_2_ are observed (Fig. 5c). This tells us that stacked ReS_2_/WS_2_ heterostructures have formed and this is attributed to the preferential growth of WS_2_ on Au and then the subsequent growth of ReS_2_ on WS_2_(001) as discussed above. The data confirms that Re atoms are adsorbed on the WS_2_(001) surface enabling nucleation and growth of ReS_2_. In addition, it should be noted that the reaction temperature of 900 °C is very crucial for the dominance of the twinned growth behaviour in the process (Supplementary Fig. 7).

Finally, we demonstrate the hydrogen evolution reaction activity for our ReS_2_/WS_2_ twinned vertically stacked heterostructures (Supplementary Fig. 8). The data show that our twinned heterostructures exhibit superior hydrogen evolution reaction activity as compared to pure WS_2_ and own great potential as a catalytic material. Besides, our ReS_2_/WS_2_ twinned vertical heterostructures also show better transfer performance than individual ReS_2_ (Supplementary Fig. 9). Furthermore, the strategy developed here is versatile as a general method for fabricating other TMDCs heterostructures. For example, MoS_2_/WS_2_ vertical heterostructures could be fabricated using Mo-W alloy foils in place of the W–Re alloy foils during the growth process (Supplementary Fig. 10).

In summary, the use of a W–Re alloy foil as a supply source of Re and W atoms, along with the difference in the adsorption energies of Re and W atoms on Au(111) surfaces allows us to demonstrate the possibility of twinned growth between 2D nanomaterials. Importantly, the presented strategy is not limited to ReS_2_ and WS_2_, which also allows the fabrication of other TMDCs vertical heterostructures such as MoS_2_/WS_2_. Besides, the strategy can be extended to any 2D material whose precursor possesses an appropriate adsorption energy at the required surface. A key factor is the correct choice of the reaction temperature to enable the selective growth of one nanomaterial over another. This type of 2D twinned vertically stacked heterostructures has a 100% overlap and their crystal sizes are one order of magnitude larger than previous reports. The simplicity of the process may be expanded to construct other vertically stacked or in-plane 2D heterostructures, thus, advancing research in the emerging field of 2D van der Waals heterostructures as well as the fundamental understanding of the nucleation and growth of TMDCs.

## Methods

### The growth of ReS_2_/WS_2_ twinned vertical heterostructures

To achieve the one-step growth of ReS_2_/WS_2_ twinned vertically stacked heterostructures, we utilized a re-solidified Au substrate in which Re and W atoms had been dissolved in. To prepare the special substrate, a piece of Au wire was placed on W–Re alloy foil. Under the protection of an Ar/H_2_ atmosphere, the Au spread evenly over the entire foil by annealing at 1,100 °C for ∼10 min which also allows Re and W atoms diffuse into the Au lattice. After that, the temperature was decreased to 900 °C for the CVD growth reaction. The simultaneous growth of ReS_2_/WS_2_ layers forming a vertically stacked heterostructure starts upon the introduction of H_2_S into the system for 10 min. This simple, scalable synthesis process is a direct CVD growth approach with no intermediate transfer steps.

### Characterization

Scanning electron microscope images were taken by Zeiss Sigma. Optical images were taken with an optical microscope (Olympus DX51). Raman spectroscopy and photoluminescence with an excitation wavelength of 532 nm were carried out using a Renishaw inVia. The atomic force microscope images were collected on an NT-MDT Ntegra Spectra. The ReS_2_/WS_2_ was transferred onto the 300 nm SiO_2_/Si for such measurements. X-ray photoelectron spectroscopy was performed on a Thermo Scientific, ESCALAB 250Xi. The measuring spot size was 500 μm and the binding energies were calibrated by referencing the C 1 s peak (284.8 eV). The TEM images were taken with an aberration corrected, high-resolution TEM (AC-HRTEM, FEI Titan^3^) operating at 80 kV. X-ray diffraction measurements were performed using a Rigaku MiniFlex600 with Cu–Ka radiation over the range of 2*θ*=10∼80°.

### DFT calculations

The spin polarized density functional theory calculations were performed using the DMol3 (ref. 32) module in the Materials Studio software (Bio Accelrys). Exchange-correlation functional was based on Perdew-Burke-Ernzerh (PBE) within the generalized gradient approximation. The core electrons were treated with DFT semi-core pseudopotentials. The optimized lattice constant of Au is 4.180 Å, in good agreement with the experimental value of 4.078 Å. The Au(111) facet was modelled by an unreconstructed 4 × 4 surface slab, which contains four atomic layers with a vacuum region of ∼20 Å. The self-consistent-field (SCF) convergence criterion was set to be <10^−5^ Hartree and the force convergence was set to be lower than 0.002 Hartree per Å for all the optimizations of the adsorptions. In particular, for the optimization of the adsorption of Re atoms and W atoms on Au(111) facets, the bottom two layers of Au atoms were fixed at their bulk positions. A 5 × 5 × 1 k-point mesh was used to sample the Brillouin zone of the supercells. The adsorption energy of a X (X=Re or W) atom on the surfaces of M (M=Au(111) or WS_2_(001)) substrate is defined as *E*_ads_^X, M^=E(M)+E(X)−E(M−X).

### Data availability

The data that support the findings of this study are available from the corresponding author upon request.

## Additional information

**How to cite this article:** Zhang, T. *et al*. Twinned growth behaviour of two-dimensional materials. *Nat. Commun.* **7,** 13911 doi: 10.1038/ncomms13911 (2016).

**Publisher's note:** Springer Nature remains neutral with regard to jurisdictional claims in published maps and institutional affiliations.

## Supplementary Material

Supplementary Information Supplementary Figures.
