# Supplementary material for: Twinned growth behaviour of two-dimensional materials
Source: Nat Commun. 2016 Dec 20;7:13911. doi: 10.1038/ncomms13911 (PMC5187448; doi:10.1038/ncomms13911)
Supplement: Supplementary Information — Supplementary Figures. [file ncomms13911-s1.pdf]

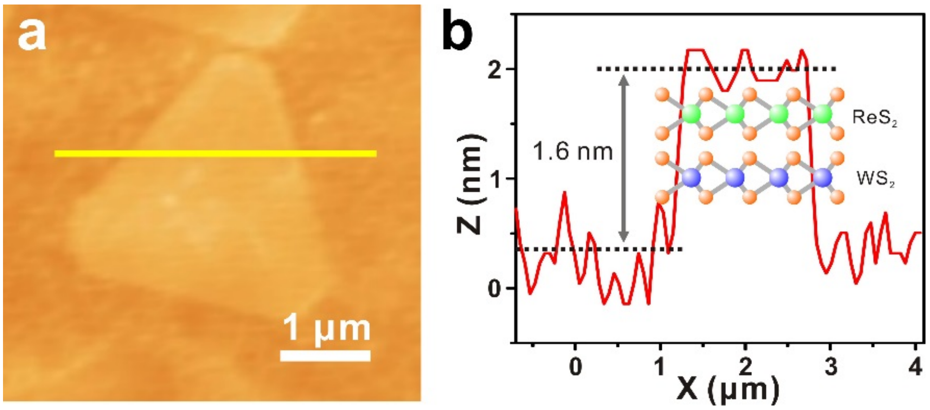

Supplementary Figure 1: AFM image of a twinned ReS_2/WS_2 crystal on Si/SiO_2. (a) AFM height topographies of a triangle ReS_2/WS_2 crystal. (b) Height profile along the yellow line in (a), showing the height difference is around 1.6 nm as approaching from the substrate to the bilayer heterostructure.

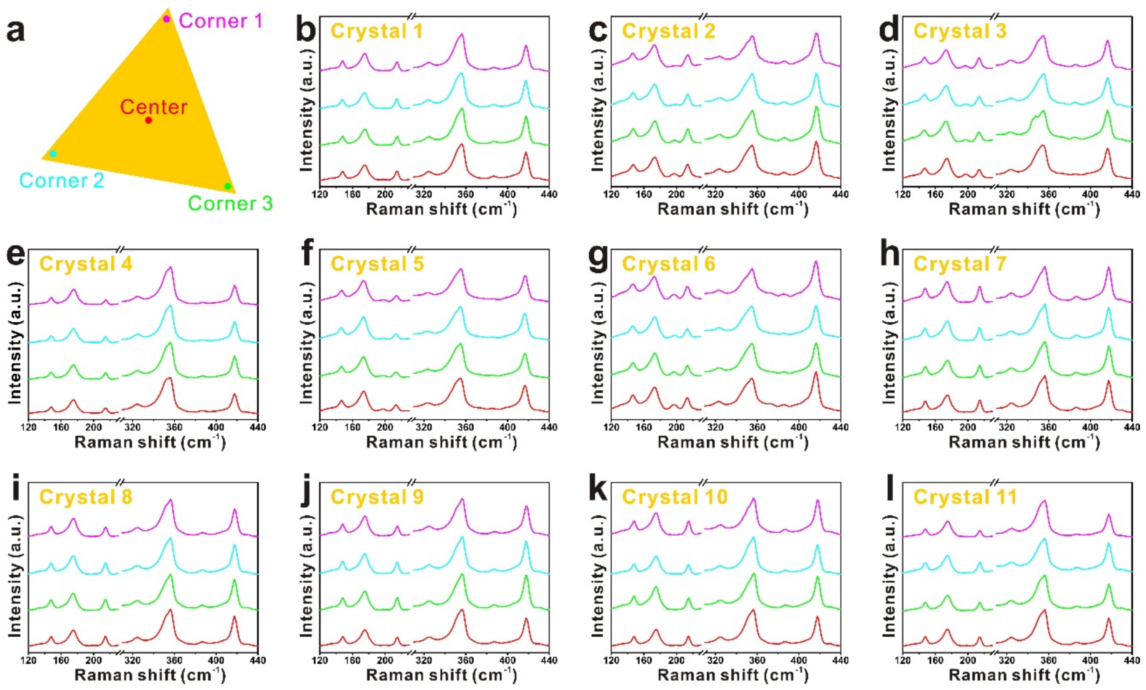

Supplementary Figure 2: Raman spectra of 11 randomly selected ReS_2/WS_2 crystals. (a) Schematic diagram showing the positions at the center and three corners of each triangle crystal where the laser spot is focused on, with colors corresponding to where each Raman spectrum was collected. (b–l) Raman spectra taken from the center and three corners of 11 randomly selected ReS_2/WS_2 crystals.

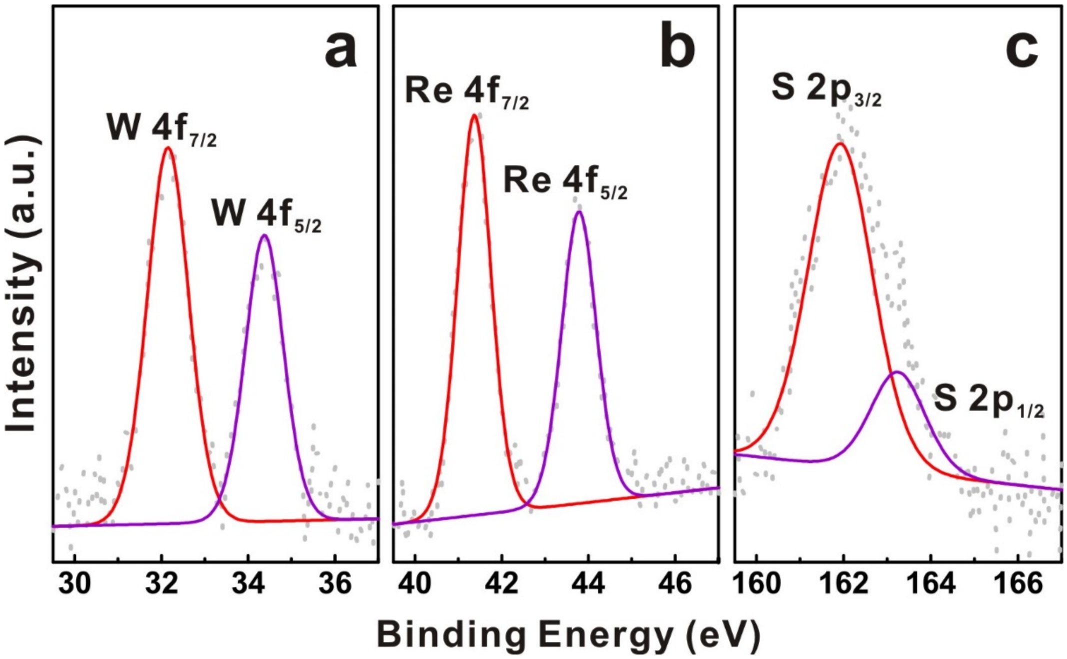

Supplementary Figure 3: XPS spectra of ReS_2/WS_2 heterostructures. (a) The two peaks at 32.1 eV and 34.2 eV are attributed to the W $4f_{7/2}$ and W $4f_{5/2}$ levels, respectively. (b) The binding energy at 41.4 eV and 43.9 eV can be assigned to the Re $4f_{7/2}$ and Re $4f_{5/2}$ levels, respectively. (c) The binding energy at 162.0 eV and 163.2 eV can be assigned to the S $2p_{3/2}$ and S $2p_{1/2}$ levels, respectively.

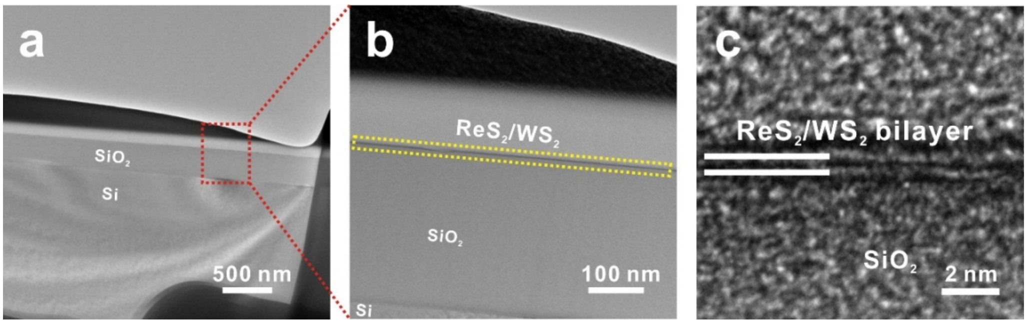

Supplementary Figure 4: Cross-sectional TEM images of ReS_2/WS_2 heterostructures. (a) Low-magnification TEM image of the ReS_2/WS_2 cross-sectional sample. (b) Zoom-in image of the region marked (red) in a, in which ReS_2/WS_2 heterostructure could be obviously seen on the top of the SiO_2 layer. (c) Cross-sectional HRTEM image of the interface between ReS_2/WS_2 bilayer structures.

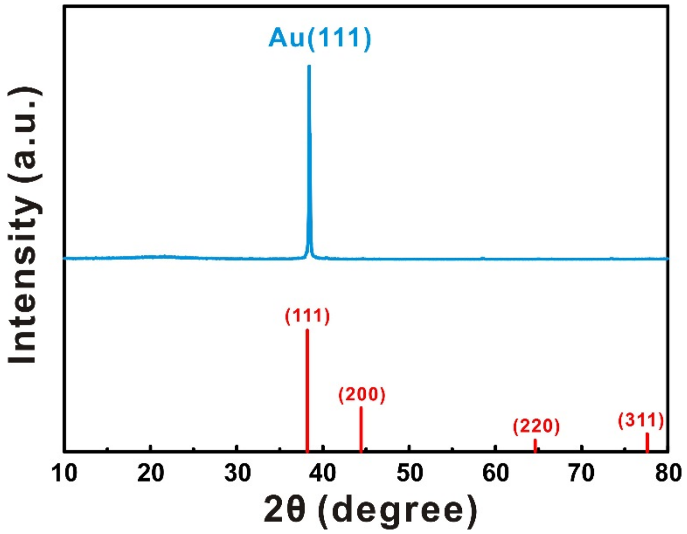

Supplementary Figure 5: XRD pattern captured from the sample.

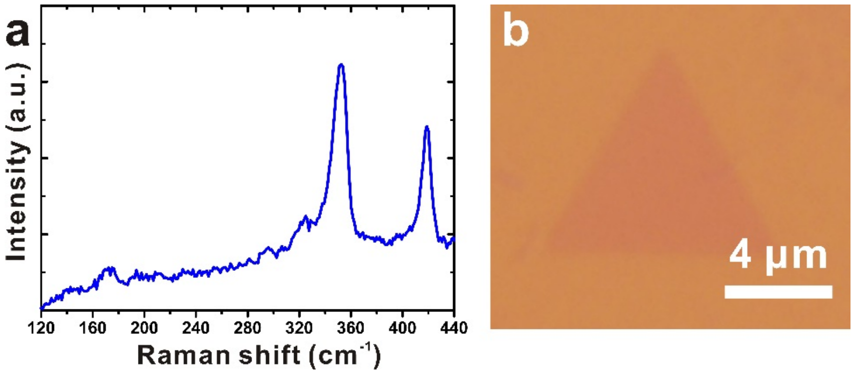

Supplementary Figure 6: Characterization of WS_2 synthesized by using W foil as the support base. (a) Raman spectrum of the as-grown WS_2 crystal on Au foil. (b) OM image of a transferred triangle WS_2 crystal on 300 nm Si/SiO_2 substrate.

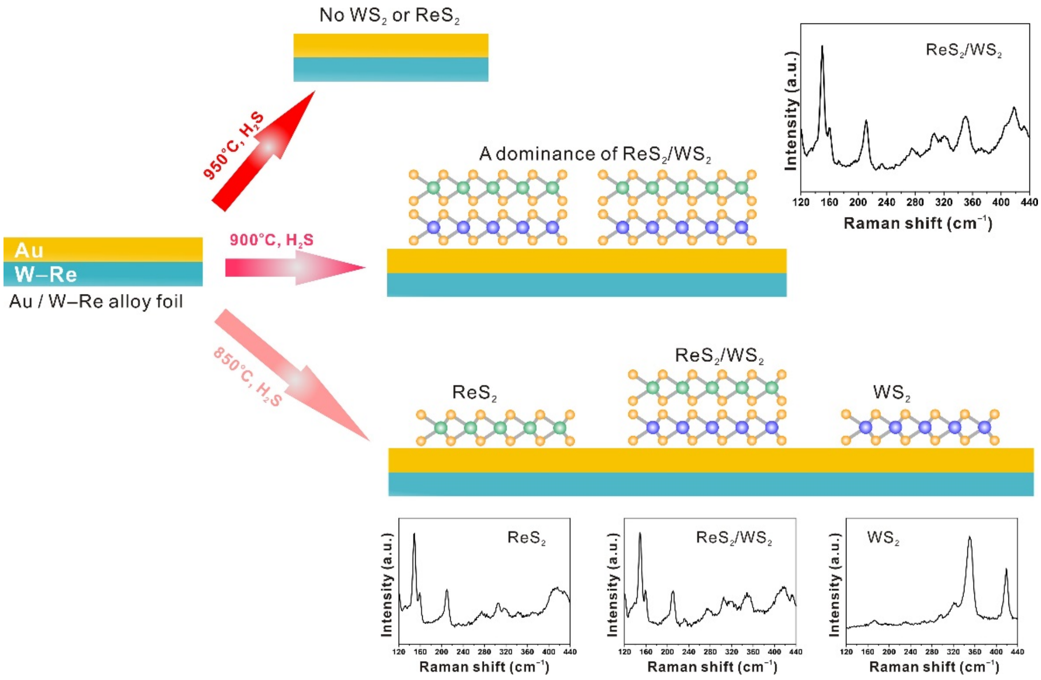

Supplementary Figure 7: Scheme and Raman data of the samples after reacting under three typical growth temperatures, which illustrate the influence of the reaction temperature on the twinned growth of ReS_2 and WS_2. After reacted at 900°C, a dominance of ReS_2/WS_2 vertical heterostructures was found on Au, which is attributed to the preferential growth of WS_2 on Au and then the subsequent growth of ReS_2 on WS_2(001) as discussed in the main text. However, with growth temperature increased to 950°C, no WS_2 or ReS_2 was obtained on the substrate, which is due to that neither W atom nor Re atom can be adsorbed on the surface at this temperature. On the contrary, while reducing the growth temperature to 850°C, both individual WS_2 and individual ReS_2 with the addition of ReS_2/WS_2 vertical heterostructures could be found on the substrate, which is because that at this temperature both W atom and Re atom can be adsorbed on the Au surface. From the above we can conclude that, the correct choice of an appropriate reaction temperature is very crucial for the dominance of the twinned growth behavior.

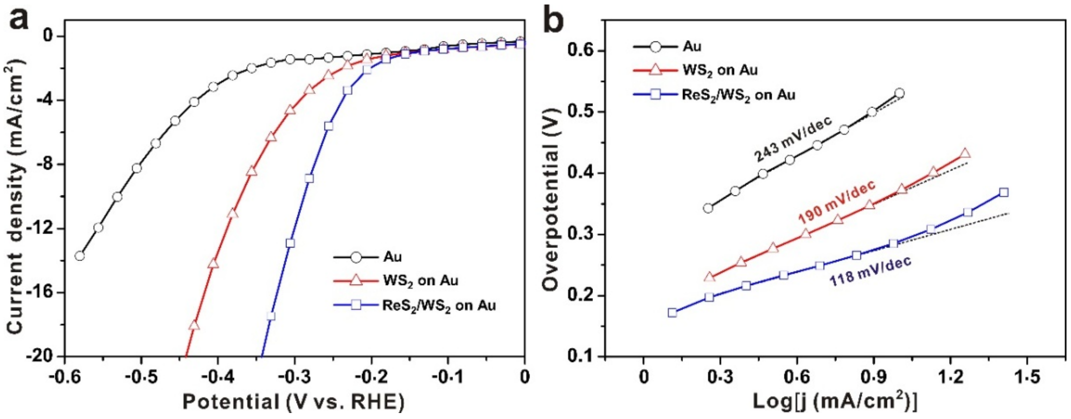

Supplementary Figure 8: Electrochemical HER catalytic activity of Au (black), WS_2 on Au (red) and ReS_2/WS_2 twinned vertical heterostructures on Au (blue) Transition metal dichalcogenides (TMDCs) have been studied as hydrogen evolution reaction (HER) catalysts. A question remains however whether the twinned TMDCs vertically-stacked heterostructures exhibit HER activity. To answer this, HER catalytic performances of both the pure WS_2 specimens and the twinned ReS_2/WS_2 heterostructures were measured with a CHI 760e electrochemistry workstation. A conventional three-electrode cell configuration was employed. A saturated calomel electrode (SCE) was used as the reference electrode, and platinum foil was used as the counter electrode. A 0.5 M H_2SO_4 solution was used as the electrolyte. All polarization curves were iR-corrected. The reference electrode was calibrated against the reversible hydrogen electrode (RHE). All the potentials reported in our work were converted according to E(*vs* RHE) = E(*vs* SCE) + 0.270 V. The Tafel slope for ReS_2/WS_2 vertical heterostructures is 118 mV dec^{-1}, which is much better than that found for pure WS_2 on Au (190 mV dec^{-1}). We suspect that the higher HER activity comes from a greater number of catalytically active sites or the unique structure at the edges of the twinned heterostructures.

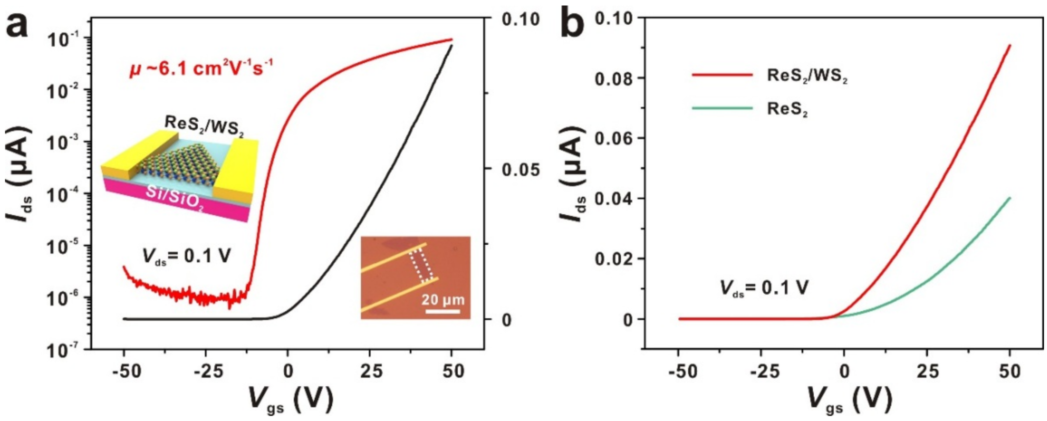

Supplementary Figure 9: Electrical properties of a twinned ReS_2/WS_2 crystal and its comparison with that of an individual ReS_2. To illustrate the electronic application of our twinned TMDCs vertical heterostructures, we constructed back-gate field-effect transistors (FETs) based on individual ReS_2 as well as ReS_2/WS_2 vertical heterostructures by electron beam lithography and electron beam deposition. All the transfer characteristics were measured at room temperature in atmosphere, with the back-gate voltage sweeping from −50 to 50 V. Typical plot of gating voltage versus source/drain current of a twinned ReS_2/WS_2 crystal is demonstrated in Figure 9a. From the transfer characteristic curve, the mobility of our ReS_2/WS_2 bilayer vertical heterostructure is calculated to be about 6.1 $cm^2\ V^{-1}\ s^{-1}$. From the comparison of I_{ds}–V_{gs} curves of back-gate FET devices based on individual ReS_2 and ReS_2/WS_2 vertical heterostructure (Figure 9b), we could find that our ReS_2/WS_2 vertical heterostructures have exhibited better transfer performance than individual ReS_2, demonstrating the great potential of our twinned TMDCs vertical heterostructures in electronic applications.

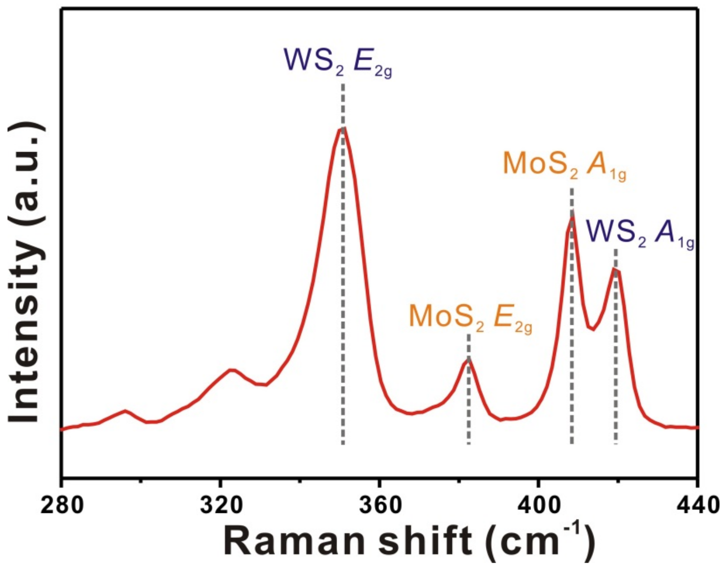

Supplementary Figure 10: Raman spectra of the twinned MoS_2/WS_2 vertical heterostructures. The twinned MoS_2/WS_2 vertical heterostructures could also be obtained in the same method except that Mo–W alloy rather than W–Re alloy was used as the support substrate.
